# The emerging role of neutrophil extracellular traps in ulcerative colitis

**DOI:** 10.3389/fimmu.2024.1425251

**Published:** 2024-08-07

**Authors:** Dan Long, Chenhan Mao, Yin Xu, Ying Zhu

**Affiliations:** ^1^ Department of Gastroenterology, The First Hospital of Hunan University of Chinese Medicine, Changsha, Hunan, China; ^2^ Affiliated Hospital of Integrated Traditional Chinese and Western Medicine, Nanjing University of Chinese Medicine, Nanjing, Jiangsu, China

**Keywords:** ulcerative colitis, neutrophils, neutrophil extracellular traps, PAD4, inflammation, immunology

## Abstract

Ulcerative colitis (UC) is characterized by chronic non-recessive inflammation of the intestinal mucosa involving both innate and adaptive immune responses. Currently, new targeted therapies are urgently needed for UC, and neutrophil extracellular traps (NETs) are new therapeutic options. NETs are DNA-based networks released from neutrophils into the extracellular space after stimulation, in which a variety of granule proteins, proteolytic enzymes, antibacterial peptides, histones, and other network structures are embedded. With the deepening of the studies on NETs, their regulatory role in the development of autoimmune and autoinflammatory diseases has received extensive attention in recent years. Increasing evidence indicates that excess NETs exacerbate the inflammatory response in UC, disrupting the structure and function of the intestinal mucosal barrier and increasing the risk of thrombosis. Although NETs are usually assigned a deleterious role in promoting the pathological process of UC, they also appear to have a protective role in some models. Despite such progress, comprehensive reviews describing the therapeutic promise of NETs in UC remain limited. In this review, we discuss the latest evidence for the formation and degradation of NETs, focusing on their double-edged role in UC. Finally, the potential implications of NETs as therapeutic targets for UC will be discussed. This review aims to provide novel insights into the pathogenesis and therapeutic options for UC.

## 1 Introduction

Ulcerative colitis (UC) is a complex chronic inflammatory bowel disease (IBD) affecting the mucosa of the rectum and colon. Its main clinical symptoms are recurrent diarrhea, mucopurulent bloody stool, and may be accompanied by extra-intestinal manifestations of varying degrees of severity (1). UC is characterized by recurrent episodes and remissions, which may result in prolonged and burdensome complications that seriously affect patients’ quality of life. A substantial body of evidence suggests that multiple factors are involved in the pathogenesis of UC, including autoimmune disorders, genetic susceptibility, defects in the intestinal epithelial mucosal barrier, and imbalances in the gut microbiota (2–4). The main pathological change in UC is an abnormal mucosal immune and inflammatory response, the main features of which include pro-inflammatory cytokines, oxygen free radicals, nitrogen production, and activation of inflammatory cells. Among them, neutrophils play a key role in intestinal homeostasis (5).

Neutrophils, as one of the most important immune cells in innate immunity, are also the body’s first line of defense against external microorganisms. It mediates antimicrobial activity through phagocytosis and degranulation, resulting in immune defense and killing of pathogens. In addition, activated neutrophils release neutrophil extracellular traps (NETs), which are complex networks comprised of DNA, histones, and granule proteins (6). NETs effectively capture pathogens and secrete antimicrobial proteins to kill them (7, 8). On the other hand, over-activated NETs may further amplify the inflammatory response, leading to tissue damage (9). Apart from infectious diseases, NETs have successively been reported to play a significant role in numerous non-infectious diseases, such as autoimmune diseases (10, 11), cancers (12–15), cardiovascular diseases (16–20), and so on. UC, an autoinflammatory disease, has not been fully elucidated in terms of its specific pathogenesis. The potential role of NETs in the pathogenesis of UC is a relatively new area of research. Here, we focus on the latest evidence for the formation and degradation of NETs, the mechanisms associated with their involvement in UC pathophysiology, and their potential role in UC therapeutic targets.

## 2 The role of neutrophils in UC

Neutrophils are the most abundant immune cells in the body, accounting for approximately 70% of peripheral blood leukocytes. Neutrophils are known for their rapid recruitment to sites of infection or tissue damage to accommodate pathogens. They can activate pathways that ultimately facilitate sustained inflammation reduction and mucosal healing (21–23). However, persistent activation and over-recruitment of neutrophils is a common feature of numerous inflammatory diseases. Neutrophils produce inflammatory factors and large amounts of reactive oxygen species (ROS), disrupt the intestinal epithelial mucosal barrier, recruit and activate other immune cells, and activate redox-sensitive inflammatory pathways (24–27). Neutrophil infiltration is associated with disease activity in UC (21, 28). Neutrophils are a widely used and reliable component of the disease scoring system for UC (29).

Neutrophils exhibit a dual role in UC, either favoring the abatement of inflammation or being deleterious when over-activated, leading to collateral tissue damage. In other words, both functional defects and hyperreactivity of neutrophils contribute to intestinal inflammation, and functional neutrophils are essential to maintain intestinal homeostasis.

## 3 Neutrophil extracellular traps

In 1996, Takei et al. (30) first discovered that neutrophils exhibit special morphological changes such as the dissociation of lobulated nuclei and rupture of nuclear and cell membranes under the stimulation of phorbol myristate acetate (PMA). In 2004, Brinkmann et al. (6) defined this new type of specific cell death, different from apoptosis and necrosis, as NETs by further validation. With subsequent studies, NETs are thought to have a DNA backbone in which a variety of active proteins are embedded, including histones, cathepsin G (CG), neutrophil elastase (NE), matrix metalloproteinase 9 (MMP-9), myeloperoxidase (MPO), calprotectin and other granule proteins, protein hydrolases, antimicrobial peptides, histones, etc. Although the NET proteome composition is fairly stable, it may be enriched with different protein components depending on the stimulus received (31).

### 3.1 NET formation

The generation of NETs by neutrophils is regulated by neutrophil intrinsic and extrinsic factors and pathways (32). The release of NETs occurs through a cell death process named NETosis (33). It is triggered by a variety of stimulants such as bacteria, viruses (34–36), fungi (37), activated platelets (38–40), immune complexes, cytokines, and chemokines (41). Studies have shown that the mechanism of NETs formation may vary depending on the initial stimulus that activates the neutrophils (42). There are two main modes of NET formation: lytic or suicidal NET formation and nonlytic or vital NET formation (43, 44).

#### 3.1.1 Lytic NETs formation

**Figure 1:**
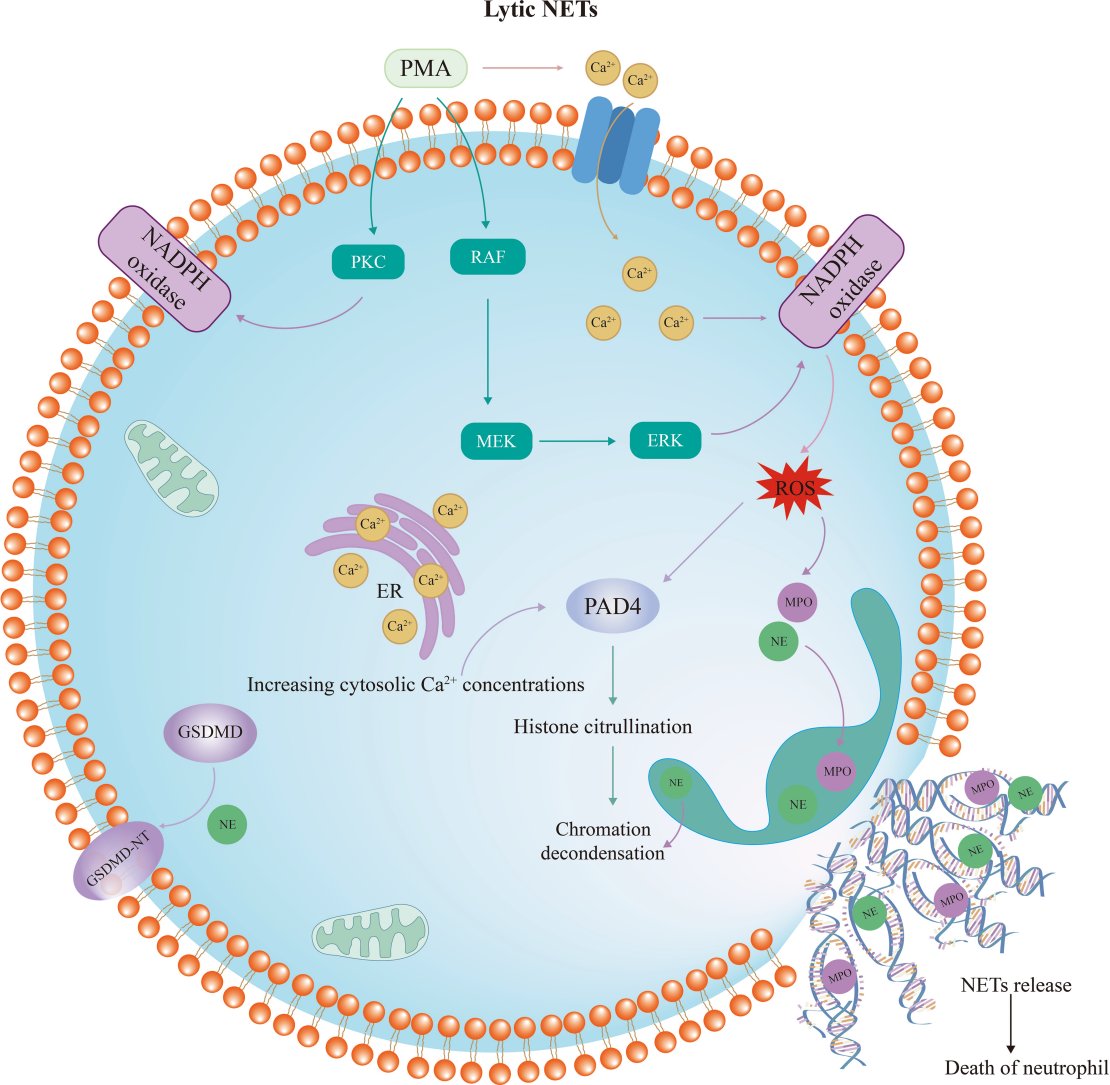
Diagram of lytic neutrophil extracellular trap formation. The RAF/MEK/ERK pathway and protein kinase C are activated by PMA, resulting in the phosphorylation of NADPH oxidase and ROS formation. This process depends on high calcium concentrations. Subsequently, PAD4 is activated and NE and MPO are translocated from the azurophilic granules to the nucleus. NE, MPO and PAD4 lead to histone citrullination and chromatin decondensation. Upon rupture of the nuclear membrane, the decondensed chromatin enters the cytoplasm and mixes with granular proteins. Finally, the cytoplasma membrane ruptures and the modified chromatin is released from neutrophils, marking the completion of NETosis. PMA, phorbol myristate acetate; PKC, protein kinase C; NADPH, nicotinamide adenine dinucleotide phosphate; ROS, reactive oxygen species; NE, neutrophil elastase; MPO, myeloperoxidase; PAD4, peptidyl arginine deiminase-4; GSDMD, Gasdermin D.

Lytic NET formation involves morphological changes in neutrophils, where activated neutrophils become flattened, lose their nucleoli, have their chromatin decondensed, and die after NETs are formed (summarized in **Figure 1**). In contrast to other cell death processes, such as apoptosis, necrosis, or pyroptosis, chromatin decondensation is the main defining feature of NETosis. NETs are generated by neutrophils stimulated by bacteria, fungi, cytokines, lipopolysaccharide (LPS) and PMA, with PMA being the most inducible (45). After stimulation with PMA or IL-8, calcium is released from the endoplasmic reticulum (ER), followed by the entrance of extracellular calcium through channels in the cellular membrane (46–49). As a result, the intracellular calcium concentrations increase in activated neutrophils (50). Interestingly, the activation of peptidyl arginine deiminase-4 (PAD4) depends on high levels of intracellular calcium concentration (51). As is well known, PAD4 is a key driver of NETosis, which causes histone citrullination and thus induces chromatin decondensation process (52, 53). Citrullinated histone H3 (Cit-H3) is generally considered as a specific marker of NET (54–57).

Apart from high intracellular calcium concentration, the mechanism of NETosis is closely related to nicotinamide adenine dinucleotide phosphate (NADPH) oxidase. PMA stimulation leads to the activation of the RAF/MEK/ERK pathway and protein kinase C (PKC), which in turn contributes to the phosphorylation of NADPH oxidase and the production of ROS (45). Then, NADPH oxidase and ROS complexes induce the translocation of NE and MPO from neutrophil granules into the nucleus (58), along with the activation of PAD4 (33). Although MPO does not directly decondense chromatin in isolated nuclei or degrade histones *in vitro*, it was demonstrated to augment chromatin decondensation mediated by NE (59). It is reported that MPO binds to chromatin and activates NE in small azurophilic structures visible *in vitro* and *in vivo*. NE disrupts actin filaments in the cytoplasm while translocating to the nucleus along with MPO (58). In the nucleus, serine proteases and NE degrade histones, further promoting chromatin decondensation. Upon rupture of the nuclear membrane, the decondensed chromatin enters the cytoplasm and mixes with granular proteins to form NET (33). Finally, the cell membrane ruptures, releasing NET and causing neutrophil death. This process is dependent on NADPH oxidase to promote ROS production and occurs slowly, typically taking three to four hours.

Interestingly, emerging evidence suggests that Gasdermin D (GSDMD), which is often considered an execution factor in pyroptosis, also plays a crucial role in cell lysis and NET release (60, 61). Inhibition of GSDMD with disulfiram or genic deletion has been shown to eliminate NET formation (62). In neutrophils, GSDMD is usually cleaved and activated by two pathways. When initiated by classical stimulus such as PMA or extracellular pathogens, NE converts GSDMD to the active form GSDMD-NT, which mediates the formation of pores in the nuclear, granular, and plasma membranes and enhances the release of NE and other granular components (60, 63). Additionally, it is well known that GSDMD could be cleaved by caspases (64). When stimulated by LPS or cytosolic Gram-negative bacteria, GSDMD is activated by caspases. Among them, Caspase-11 is required for GSDMD-dependent NET formation (61). It has been reported that the specific deletion of Caspase-11 in neutrophils significantly inhibited NET formation (65). Caspase-11 and GSDMD are not only required for plasma membrane rupture in neutrophils during the final stages of NET release, but are also essential for the early features of NETosis, including nuclear foliation and DNA amplification, which are mediated by nuclear membrane permeabilization and histone degradation induced by Caspase-11 and GSDMD (61).

#### 3.1.2 Nonlytic NETs release

**Figure 2:**
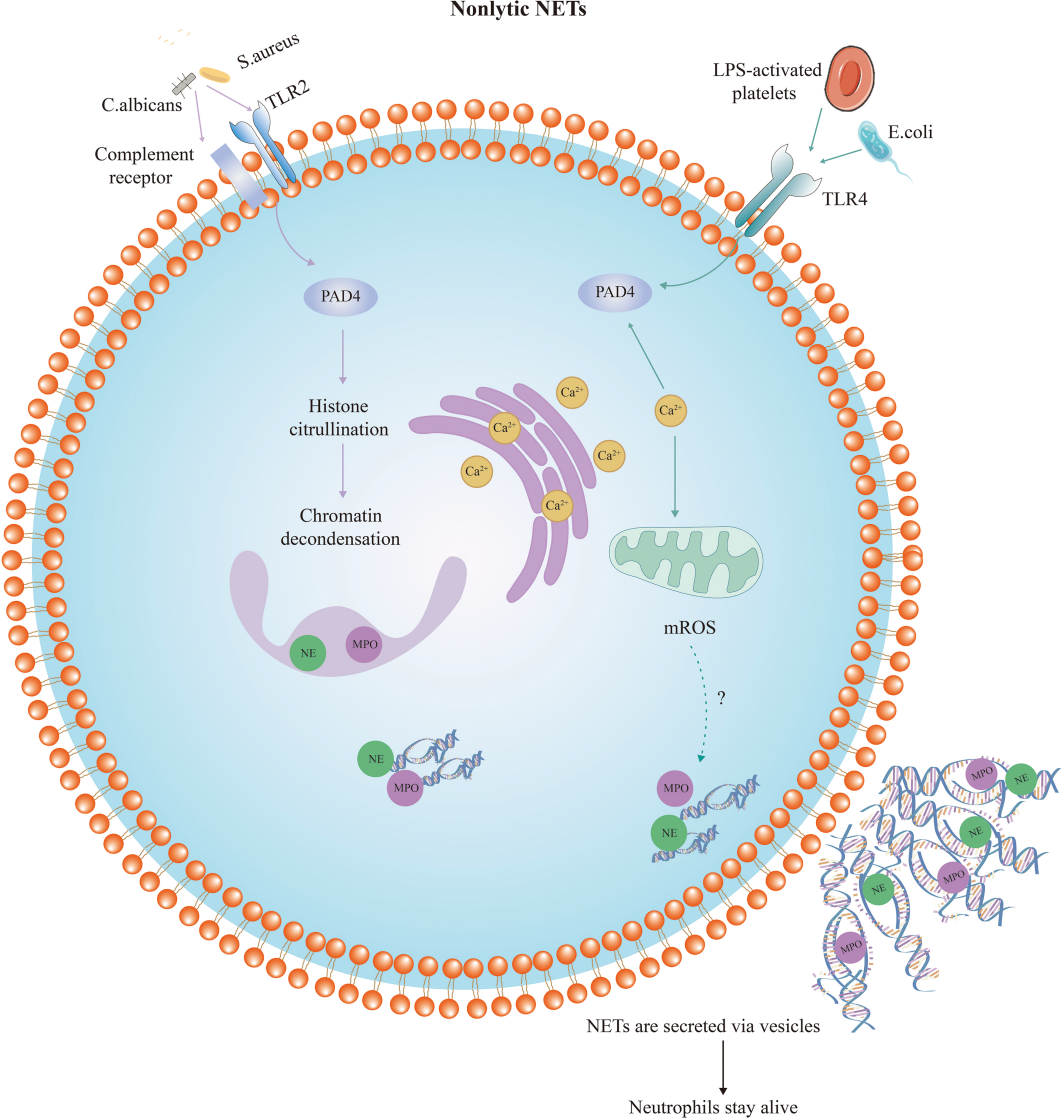
Diagram of nonlytic neutrophil extracellular trap formation. Compared with suicidal NETosis, nonlytic NET formation is completed in a shorter period of time and may occur in the absence of NADPH oxidase and ROS. This form of NET formation is also triggered by a sudden elevation of intracellular calcium levels. Nonlytic NET formation is initiated by stimuli such as *Staphylococcus aureus* activating TLR2, or *Escherichia coli* activating TLR4. PAD4 is activated and NE and MPO translocate to the nucleus to stimulate chromatin decondensation. Decondensed chromatin decorated with granulin and histones is packaged in vesicles budding from the nucleus. Then, these vesicles are expelled from intact neutrophils and form NETs near the neutrophils. Thus, the neutrophils remain intact and maintain their function.TLR, Toll-like receptor; LPS, lipopolysaccharide; NE, neutrophil elastase; MPO, myeloperoxidase; PAD4, peptidyl arginine deiminase-4.

A nonlytic and NADPH-oxidase-independent mechanism of NET formation was subsequently discovered, which appears to maintain neutrophil integrity and viability by actively releasing DNA-containing vesicles (33, 43). Unlike lytic NET formation, this process releases NETs less than an hour after stimulation. Nonlytic NET release plays a more important role in regulating pathogen infection. This form of NET formation is triggered by a sudden elevation of intracellular calcium that results in the expulsion of nuclear chromatin and granule proteins, causing anucleate cytoplasm to remain capable of migration and phagocytosis (summarized in **Figure 2**). *Staphylococcus aureus* and *Candida albicans* activate Toll-like receptor 2 (TLR2) and complement receptors, respectively, while platelets activated by LPS and Escherichia coli activate TLR4 (66, 67). TLRs and complement receptors activate PAD4 and activated PAD4 triggers histone citrullination, resulting in chromatin decondensation. The decondensed chromatin enters into the cytoplasm and binds granzymes such as MPO, NE, and other proteins, eventually releasing into the extracellular space. However, instead of plasma membrane disruption through vesicle release, protein-modified chromatin is secreted through vesicles. The nuclear and cellular membranes remain intact during this process, and neutrophil viability and functions such as phagocytosis and chemotaxis are unaffected (44). Although this pathway is not dependent on NADPH oxidase-derived ROS, a recent study has demonstrated that calcium ion carriers induce the production of mitochondrial ROS (68). After being stimulated by granulocyte-macrophage colony-stimulating factor or LPS, neutrophils release mitochondrial DNA (mtDNA) mixed with granule proteins (69). Additionally, it has been proven that deletion of mtDNA results in a significant reduction in NADPH-oxidase-independent NET production, suggesting that mitochondria are also important in NET formation (70). The specific mechanism of mitochondria in this process requires further study.

### 3.2 NET degradation

Although numerous researchers have been focusing on the molecular mechanisms of NET formation, little is known regarding NET degradation. Actually, the balance between the formation and degradation of NETs is critical for maintaining NET homeostasis. Since DNA is the main component of NETs, DNA degradation enzymes play an instrumental role in NET degradation. In autoimmune diseases, reduced Deoxyribonuclease (DNase) activity is strongly correlated with NET accumulation. DNase is regarded as an essential enzyme for degrading NET *in vivo* by cleaving circulating free DNA (71). DNase consists of two families, DNase I and DNase II. The DNase I family is composed of four members including DNase I, DNase1L1, DNase1L2, and DNase1L3, whereas the DNase II family consists of DNase II α and DNase II β (72). The kinetics of hydrolysis are influenced by the structure and sequence of the DNA substrate. Double-stranded DNA (dsDNA) is cleaved by DNase I 100-500 times faster than single-stranded DNA (ssDNA) (73, 74). It was reported that DNase1 inhibitors or anti-NET antibodies preventing DNase1 entry into NETs both resulted in impaired NET clearance (71). Li et al. revealed that the downregulation of DNase1L3 expression is a critical cause of impaired DNA clearance in NETs (75). In addition, the accumulation of NETs and their components is associated with the formation of anti-dsDNA, anti-histones, and anti-nucleosome antibodies, which are recognized as pathogenic factors in systemic lupus erythematosus (SLE) (76).

Apart from the DNase family, other enzymes have been revealed to disrupt the structure of NETs, such as 3′-exonucleases (TREX1 and TREX2) (77, 78). Interestingly, macrophages and dendritic cells were found to exert an important role in the intracellular and extracellular degradation of NETs. NET degradation mediated by extracellular DNase1L3 was observed in dendritic cells, while intracellular degradation of NETs by macrophages relied on the function of TREX1 (77). A growing number of researches emphasized the prominent role of macrophages in NET degradation (79, 80). Farrera et al. confirmed that macrophages effectively removed NETs by taking up extracellular DNA (79). Macrophage-secreted DNase I facilitates this process by extracellularly digesting large fragments of NETs, whereas complement factor 1q (C1q) promotes NETs opsonization. NETs phagocytosed by macrophages are degraded in the lysosomal compartment. Interestingly, a recent study indicated that the polarization of macrophages affected their ability to degrade NETs (81). In particular, proinflammatory polarization promotes NET degradation through enhanced macropinocytosis.

### 3.3 Functions of NETs

NETs were originally described as host defense mechanisms for trapping or killing neutrophilic pathogenic microorganisms. NETs encapsulate proteins such as histone, NE, MPO, and CG, which trap pathogens and prevent them from spreading to secondary sites of infection, exerting anti-bacterial, fungal, and viral effects (6, 31). In neutrophils, NADPH is produced by glucose 6-phosphate in the oxidation branch of the pentose phosphate pathway. A recent study by Ulrich and colleagues demonstrated that low levels of NADPH and ROS in patients with defects in glucose-6-phosphate dehydrogenase caused impaired formation of NETs and greatly increased the risk of bacterial infection (82). Indeed, impairing NET formation might exacerbate inflammation and worsen conditions (7, 83). NET also modulates other immune cells. For instance, by inhibiting dendritic cell activation, it promotes the Th2 response and facilitates inflammation to subside (84). However, NET is a double-edged sword. There is growing evidence that when there is an imbalance between the formation and removal of NETs, the deposition of excess NETs in tissues and organs promotes an inflammatory response (85, 86) and drives disease progression (9, 87, 88). NETs recruit macrophages and other pro-inflammatory cells or proteins, promote the release of inflammatory factors such as IL-1β and IL-6, and activate NLRP3 inflammatory vesicles to further amplify the inflammatory response (89–91). Overall, the anti-inflammatory effects of NETs are counteracted by pro-inflammatory effects in disease. A growing body of research suggests that NETs are associated with the progression of several diseases and may serve as potential biomarkers for disease diagnosis and prognosis (92–95).

## 4 Increased NETs formation in UC

A growing number of studies have shown that NET-related proteins have enhanced expression in the inflamed colon of UC patients (96–99). Proteomic studies revealed that the abundance of NET-associated proteins in colonic mucosal samples from patients with UC was, on average, 42.2 times higher than in normal colons (100). Western blotting analysis and quantitative immunoblotting indicated that PAD4 expression was dramatically enhanced in inflamed mucosa of UC patients compared to Crohn’s disease (CD) patients or healthy individuals (98). Western blotting analysis has also shown that the expression of NE, MPO, and Cit-H3 was significantly up-regulated in colon samples from UC patients. Moreover, the double-immunofluorescence assay indicated that these three proteins were co-localized in UC mucosa. Similarly, confocal microscopy analysis also exhibited positive staining for citH3 and NE, overlapping with diffuse DNA scaffolding, confirming NET deposition in the colon of patients with active UC (101). Additionally, recent studies suggest that plasma levels of NET are significantly elevated in UC patients (101, 102). Other evidence points out that neutrophils isolated from UC patients are more likely to form NETs when stimulated *in vitro (* 98, 101).

Apart from patients with UC, it has been demonstrated that NETs and related proteins are significantly increased in a mouse model of experimental colitis (98, 103). A recent study by Li and colleagues showed elevated serum levels of cell-free DNA (cfDNA) and increased NET formation in mice with dextran sulfate sodium (DSS)-induced colitis (101). Mechanistically, LINC00668, which is highly enriched in intestinal epithelial cell (IEC)-derived exosomes, mediates the translocation of NE from cytoplasmic granules to the nucleus, thus stimulating histone cleavage and chromatin decondensation and triggering NETs release (104). Other evidence suggests that high levels of antineutrophil cytoplasm autoantibodies (ANCA) in serum induce neutrophil aggregation and foster NET generation (105).

Damage-associated molecular patterns (DAMPs) are molecules released in response to cellular stress or tissue damage and have been demonstrated to function as direct pro-inflammatory mediators (106, 107). Several researches have identified DAMPs capable of inducing NET formation in UC (108). For example, cfDNA is a DAMP capable of enhancing NET generation (109). Interestingly, cfDNA is significantly increased in the serum of UC patients as well as in murine UC models, and its plasma concentration is positively correlated with clinical severity (110, 111). cfDNA has a strong potential to trigger NETosis, resulting in endothelial cell injury (112). High-mobility group box 1 (HMGB1) is another DAMP known for its ability to promote NETs (113, 114). Recent evidence suggests that TLR4 and C-X-C motif chemokine receptor 4 (CXCR4) are specific receptors for HMGB1 (115). Notably, HMGB1 not only induces NETosis but is also involved in the extrusion of NETs. Chen et al. detected that HMGB1 was significantly higher in the inflamed colon of UC patients (116). They also demonstrated that anti-HMGB1 neutralizing-antibody improved intestinal barrier function and inflammation in DSS-induced colitis mice.

Moreover, in UC, cytokines are important mediators in inducing NETosis. As is well known, IL-1β, IL-6, tumour necrosis factor α (TNF-α), and other cytokines are expressed at relatively higher levels in the intestinal tissues of UC patients (117). These cytokines trigger macrophages and neutrophils to phagocytose and to release effector mediators. It has been demonstrated that neutrophils from UC patients produced significantly more NETs under the stimulation of TNF-α (98). In addition, NET formation was significantly reduced in colons of UC patients treated with infliximab, a TNF-α inhibitor, indicating a strong stimulatory effect of TNF-α in NETosis (98). UC patients show significantly enhanced expression of IL-8 (118, 119), a cytokine that not only stimulates neutrophil recruitment but also triggers NETosis (120, 121). Similarly, IL-6, a cytokine widely increased in UC patients and colitis mice (122, 123), serves as another potent inducer of NETosis (124, 125). However, there are still substantial gaps in the upstream mechanisms of NETosis induction in UC, which need to be further explored.

## 5 Impaired clearance of NETs in UC

As mentioned above, several autoimmune diseases have been linked to dysregulated NET clearance (126). The balance between generation and degradation of NETs is critical in UC and other autoimmune diseases. Some findings suggest that in UC, the increase in NETs may be attributed to decreased clearance rather than merely increased production (101). Li et al. (101)detected impaired degradation of NETs in the plasma of patients with active UC. DNase I activity has been reported to be significantly lower in IBD patients than in healthy individuals (127). DNase I activity is strongly negatively correlated with the serum concentration of anti-nucleosome antibodies. Impaired DNaseI-mediated NET clearance is probably related to the pathogenesis of IBD. Thus, the ability of UC patients to clear NETs and specific mechanisms deserve further research.

## 6 The double role of NETs in UC

### 6.1 The role of NETs in the pathogenesis of UC

#### 6.1.1 NETs exacerbate inflammatory response in UC

UC mainly manifests as a chronic, recurrent, non-specific inflammatory response in the intestinal mucosa. Neutrophil infiltration correlates with endoscopic severity and systemic inflammatory indices, whereas serum levels of C-reactive protein and fecal lactoferrin and calprotectin may serve as sensitive markers of intestinal inflammation (128–130). Notably, both lactoferrin and calprotectin are key components of NETs (128). NET is associated with diverse immune and inflammatory diseases, including UC, and is thought to maintain mucosal inflammation in this disease (98).

**Figure 3:**
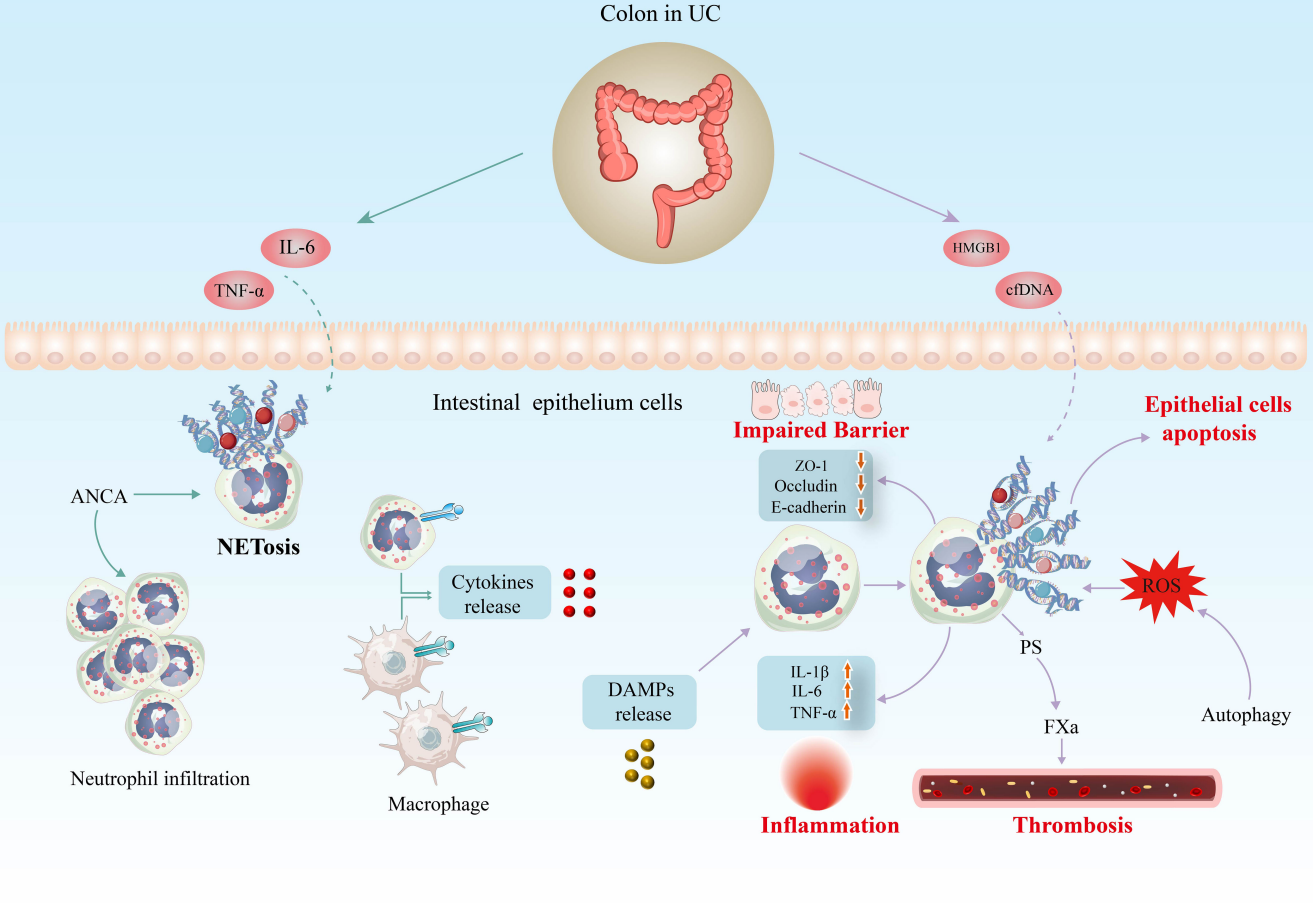
Potential implications of NETs in the pathogenesis of UC. Inflammatory factors such as IL-6 and TNF-α are potent inducers of NETosis in UC models. In addition, high levels of antineutrophil cytoplasm autoantibodies (ANCA) in serum induce neutrophil infiltration and foster NET generation. Several researches have identified DAMPs capable of inducing NET formation in UC, including cfDNA and HMGB1. Increasing evidence indicates that excess NETs exacerbate the inflammatory response in UC, disrupting the structure and function of the intestinal mucosal barrier and increasing the risk of thrombosis. IL-6, interleukin 6; TNF-α, tumour necrosis factor α; cfDNA, cell-free DNA; HMGB1, high-mobility group box 1; DAMP, damage-associated molecular pattern; ROS, reactive oxygen species; PS, phosphatidylserine.

It has also been shown *in vitro* that NETs enhance the secretion of TNF-α and IL-1β in UC lamina propria mononuclear cells by promoting the phosphorylation of ERK (98). As shown in **Figure 3**, NETs induce the release of pro-inflammatory cytokines from macrophages, thus stimulating local and systemic inflammatory responses (131). A recent study showed that NETs significantly boosted the response of monocyte-derived macrophages to low-dose LPS and increased the release of TNF-α, IL-6, and monocyte chemotactic protein-1 (MCP-1) (101). PAD4 is considered a biomarker of NETosis as its suppression or gene deficiency in neutrophils inhibits this process (132). Numerous researchers have been focusing on the critical role of PAD4 in NETs of UC. Considerable studies have revealed that PAD4 expression is significantly elevated in colonic samples from UC patients (96, 133). Immunohistochemistry analysis of paired colon sections from UC patients showed more pronounced PAD4 expression in inflamed areas compared to uninvolved mucosa from the same patients (98). Blocking the formation of NETs by PAD4 knockout alleviates clinical colitis indices, intestinal inflammation, and barrier dysfunction (134).

Increasing studies have shown that cellular autophagy may play an essential role in the formation of NETs (135–137). Inhibition of autophagy leads to reduced generation of ROS and NETs (138). Another researcher revealed that inhibition of ATG7, a critical autophagy-associated protein, suppressed autophagy and significantly reduced NET formation (139). Moreover, increased NET formation in UC patients has been demonstrated to be autophagy-dependent and is associated with increased expression of regulated in development and DNA damage response 1 (REDD1) in neutrophils. The REDD1/autophagy/NETs axis is involved in the IL-1β-driven inflammatory response in UC patients (97).

#### 6.1.2 NETs impair epithelial barrier function in UC

Impaired intestinal epithelial barrier function due to IEC injury is one of the key features of the pathophysiology of UC. Also, damage to the vascular endothelium cell results in delayed healing of the localized ulcerated mucosa, exacerbating intestinal damage. It was found that in mouse with DSS-induced colitis, the presence of NET structures was not limited to the lamina propria and epithelium, but also in the intestinal lumen (103). NETs increase bacterial translocation by increasing intestinal barrier permeability and disrupting colonic epithelial barrier function. Further studies revealed that the NET-induced deterioration of barrier function was attributed to the promotion of apoptosis in IECs (103). Compared with control mice, expression of the tight junction proteins occludin and ZO-1 as well as the adhesion junction component E-cadherin was reduced in Caco-2 monolayers treated with NETs or histones (103). NETs-associated histones alter the integrity of tight junctions and adhere to junctional proteins and induce IEC death, leading to increased intestinal epithelial permeability (140). Whether NETs are cytotoxic to endothelial cells, leading to intestinal tissue damage in UC, remains to be investigated.

#### 6.1.3 NETs and thrombotic tendency in UC

Patients with IBD face three times the risk of thromboembolic events than the general population (141). In recent years, the association between NETs and arteriovenous thrombosis has attracted widespread attention. The network structure of NETs provides a scaffold for the deposition of platelets, erythrocytes, fibrinogen, platelet adhesion factor, and other substances that favor thrombosis (142). At the same time, a number of components of NETs actively trigger platelet activation and blood coagulation. There is already considerable evidence of a link between NETs and thrombosis in IBD (101, 143, 144). Research has shown that incubation of normal platelets with NETs from patients with active UC and CD resulted in a significant 32% increase in their procoagulant activity and a 42% increase in their ability to support fibrin formation. Another study (101) revealed that NET-induced platelet activation is mediated by signaling through TLR2 and TLR4. NETs from patients with active UC trigger increased exposure to phosphatidylserine in endothelial cells in a time-dependent manner, which promotes thrombin generation as well as the production of intrinsic and exogenous FXa complexes. Researchers found a significant increase in NETs in inferior vena cava thrombosis in DSS-induced IBD mice (104). Further studies discovered that this was related to exosomes released by IECs under inflammatory conditions. The specific mechanism of NETs on the thrombosis tendency in UC deserves further in-depth study.

### 6.2 The beneficial role of NETs in UC

Although NETs are often assigned a deleterious role in promoting inflammatory responses, there is growing evidence that they exert beneficial anti-inflammatory effects in a variety of conditions. For example, during severe sepsis, NETs have proved indispensable in trapping circulating bacteria to prevent systemic infection (67). NETs have been demonstrated to promote the regression of gouty inflammation through the degradation of cytokines and chemokines by serine proteases (7). When NET formation is impaired, monosodium urate crystals induce uncontrolled production of inflammatory mediators. Hahn and other researchers have also found that NETs work through a similar mechanism to reduce inflammation in periodontitis (145). In rheumatoid arthritis, NETs inhibit IL-6 secretion from LPS-activated macrophages and induce secretion of the anti-inflammatory factor IL-10 (146).

The formation of NETs during necrotizing enterocolitis has been reported to reduce intestinal bacterial translocation and attenuate systemic inflammation (83). Conversely, the reduction of NETs may lead to increased systemic inflammation in mice, ultimately resulting in the severe consequences of bacteremia and significantly reduced survival. Another study confirmed that activation of triggering receptor expressed on myeloid cell-1 (TREM-1) clears pathogens and protects the intestinal barrier by increasing the production of NETs and IL-22 by CD177 neutrophils, which is likely to be an effective therapeutic strategy for IBD (147). In addition, a recent study showed that PAD4-deficient mice had an exacerbated course of colitis and a significant increase in rectal hemorrhage compared to controls (133). The PAD4-dependent NET formation is closely associated with the remodeling of the blood clot into a secondary immune thrombus, thereby preventing rectal hemorrhage in UC. Therefore, caution must be exercised when targeting NET as a treatment for UC.

## 7 NETs as potential therapeutic targets in UC

The higher expression of NETs in UC plays an influential part in its pathogenesis and development, so targeting NETs to treat UC has gradually become a research hotspot. The regulation of NET generation or DNA degradation appears to be two possible effective strategies in UC therapy, and a balance of these two approaches may be better for positive outcomes. Recent studies have identified a number of drugs, including phytomedicine, that suppress UC through targeting NETs, as outlined in **Table 1**.

**Table 1:** Summary of potential NETs-targeted therapies in UC.

Modulation on NETs	Targets	NETs treatment	Dose/concentration	Models and participants	Mechanism of action	References
Inhibit NETs formation	PAD4	Cl-amidine	5, 25, and 75 mg/kg/day (mice)	DSS-induced colitis mice	Inhibition of PAD activity and protein citrullination	(148)
		Cl-amidine	30 mg/kg/day (mice)	TNBS-induced colitis mice	PAD4↓, Cit-H3↓, MPO↓, TNF-α↓, IL-1β↓, IL-6↓, IL-17A↓, IFN-γ↓, IL-10↑	(149)
		Cl-amidine	30 mg/kg/day (mice)	TNBS-induced colitis mice	Cit-H3↓, MPO↓, IL-1β↓	(150)
		streptonigrin	0.4 mg/kg/day (mice)	DSS-induced colitis mice	citH3↓, LCN-2↓, TNF-α↓, IL-1β↓	(98)
		H_2_S donor	18.75 µM/kg/day (rat)	TNBS-induced colitis rat	PAD4↓, Cit-H3↓, MPO↓, NF-κB↓, HMGB1↓	(151)
		Huang Qin Decoction	500, 1000, and 1500 mg/kg/day (mice)	AOM/DSS-generated CAC mice	PAD4↓, Cit-H3↓, MPO-DNA complex↓, TNF-α↓, IL-1β↓, MMP-9↓, ZO-1↑, occludin↑	(152)
	ROS	5-aminosalicylic acid	0.005, 0.25, and 0.5 mM (cell)	Human peripheral blood neutrophil	ROS↓, superoxide↓	(153)
		Cyclosporine A	not mentioned (mice) 100 nM (cell)	DSS-induced acute colitis mice, peripheral blood neutrophil from UC patients	P53↑, G6PD↓, ROS↓, Cit-H3↓, NE↓, TNF-α↓, IL-1β↓,	(102)
		butyrate	200 mM in drinking water (mice)	DSS-induced acute colitis mice	ROS↓, CitH3↓, IL-6↓, TNF-α↓, IFN-γ↓, CXCL1↓,	(154)
	Ne	berberine	1 mg/kg/day (mice)	DSS-induced IBD mice	NE↓, MPO↓, CitH3↓	(104)
	TNF-α	infliximab	5 mg/kg at weeks 0, 2 and 6	UC patients	PAD4↓, Cit-H3↓, MPO↓	(98)
	FcRn	anti-Fc-mAb	80 and 160 μg/kg/7 days	DSS-induced UC rat	ANCA↓, PAD4↓, Cit-H3↓, MPO↓, NE↓, TNF-α↓, CRP↓	(105)
	MPO	Ferulic acid	50mg/kg/day (mice)	DSS-induced colitis mice	MPO-DNA complex↓, Il-17↓, Il-22↓	(155)
Degrade NETs	DNA matrixes	DNase I	250 U, qod (mice)	DSS-induced colitis mice; TNBS-induced colitis mice	MPO-DNA complex↓, CitH3↓, TNF-α↓, IL-1β↓, ZO-1↑, occludin↑	(103)
		DNase 1	65 U DNase 1, qod (mice)	DSS-induced colitis mice	MMP-9↓, TNF-α↓, IL-6↓, MCP-1↓, IL-1β↓, CXCL2↓, CXCL10↓	(101)
		staphylococcal nuclease	25 and 75 mg/kg/day (mice)	DSS-induced UC mice	MPO↓, NE↓, TNF-α↓, IL-6↓, IL-1β↓, ZO-1↑, occludin↑	(156)

### 7.1 PAD4 inhibitors

Theoretically, any important link in the formation of NETs may become a potential target for the treatment of UC (summarized in **Table 1**). As mentioned above, PAD4 is a key enzyme in the formation of NETs. Therefore, it is also an important target for blocking the pathological effects of NETs. At present, the most representative PAD inhibitor in the UC field is the irreversible inhibitor Cl-amidine. According to reports, Cl-amidine blocked the formation of NET structure, reduced the expression of PAD4 and cit-H3 in the colon tissue, and effectively alleviated clinical colitis index in TNBS-induced colitis mice (149). The inhibition of PAD4 mediated by CL-amidine reduces the expression of pro-inflammatory cytokines such as TNF- α, IL-1 β, and IL-6, while the expression of anti-inflammatory factor IL-10 is also upregulated (149). CL-amidine also has antioxidant consequences in a colitis mouse model, with the ability to suppress leukocyte activation and prevent colon epithelial DNA damage (157).

In addition, streptonigrin, a selective PAD4 inhibitor, has also been shown to reduce the expression of pro-inflammatory cytokines such as TNF-α and IL-1 β, as well as NETs-associated proteins, alleviating colonic inflammation (98). Interestingly, a recent study showed that H_2_S donor significantly inhibited PAD4, Cit-H3 and MPO expression in TNBS-induced colitis rats (151). It appears to exert an anti-inflammatory effect by inhibiting NET formation and downregulating NF-κB and HMGB1 expression.

### 7.2 NADPH/ROS inhibitors

Similarly, NET formation is suppressed by inhibiting the expression of proteins such as Ne (158) and ROS (153). Cyclosporine A (CsA), a well-known immunosuppressive drug, is routinely prescribed for the treatment of patients with steroid-refractory acute severe ulcerative colitis (ASUC) (159, 160). It not only suppresses IL-2 secretion by T cells, but also interferes with dendritic cell (DC) migration (161, 162). Strikingly, CsA suppresses a variety of neutrophil processes, including ROS production and NET formation (70, 163). It has been demonstrated to decrease ROS generation in isolated human neutrophils (159, 164). A recent study revealed that CsA inhibits apoptosis and migration as well as the release of ROS, MPO, and antimicrobial peptides from neutrophils in ASUC patients (165). Mechanistically, CsA inhibits Sirtuin 6 (SIRT6) expression and subsequently promotes hypoxia‐inducible factor‐1α (HIF-1α) expression in neutrophils as well as glycolysis and the tricarboxylic acid cycle to limit neutrophil overactivation, thus alleviating mucosal inflammation in ASUC patients. Recently, Xu et al. (102) identified that CsA directly reduces the activity of pentose phosphate pathway (PPP) rate-limiting enzyme G6PD via activating P53 protein and represses PPP metabolism to produce ROS, thereby reducing ROS-dependent NETs release and attenuating colitis.

### 7.3 DNase I

In addition, several studies have demonstrated that DNase I is a promising treatment for UC (101, 103). DNase I is an enzyme that dissolves reticulated DNA filaments of NET. In mice with DSS-induced colitis, DNase I improved fecal consistency and reduced fecal occult blood and rectal bleeding (103). In addition to improved intestinal inflammation, DSS mice treated with DNase I showed significantly increased expression levels of tight junction protein occludin and ZO-1. Meanwhile, the disruption of NET structure by DNase I was demonstrated to also restore the structure and function of the intestinal mucosal barrier in mice with TNBS-induced colitis, reduce the levels of pro-inflammatory cytokines, and alleviate intestinal inflammation (103).

### 7.4 Bioextracts

The treatment of autoimmune and inflammatory diseases by modulating NETs in traditional Chinese medicine (TCM) is an area of great interest. For instance, crocetin inhibits PMA-induced NETs formation, as evidenced by reduced expression of NE, PAD4, and CitH3, and alleviates symptoms in adjuvant-induced arthritis mice (166). Apart from inhibiting the activation of the nuclear factor-κB (NF-κB) and mitogen-activated protein kinase (MAPK) pathways, the mechanism involves inhibiting the formation of NETs by suppressing autophagy. Moreover, celastrol is a triterpenoid compound. Celastrol has been shown to completely inhibit neutrophil oxidative burst and NET formation induced by TNF-α. Celastrol downregulates the activation of spleen tyrosine kinase (SYK) and the concomitant phosphorylation of MAPKK/MEK and ERK, as well as the citrullination of histones (167). It suggests that celastrol probably has the potential to modulate inflammation involving neutrophils and NETs and to have therapeutic implications for autoimmune diseases. HMGB1 protein secreted by NETs promotes the release of cytokines and chemokines, causing inflammatory responses. While celastrol effectively suppresses the expression of HMGB1, NF-κB and IL-1β to reduce inflammatory pain (168). Triptolide alleviates chronic arthritis by reducing neutrophil recruitment and suppressing the expression of IL-6 and TNF-α (169). It also inhibits the expression of pro-inflammatory cytokines and NETosis in neutrophils. Furthermore, glycyrrhizin has been demonstrated to inhibit TLR9/MyD88 activation by decreasing HMGB1 expression, thereby reducing NET formation and alleviating sepsis-induced acute respiratory distress syndrome (170). Moreover, forsythiaside B improves coagulopathy in septic rats by inhibiting the formation of PAD4-dependent NETs (171). Luteolin significantly inhibits superoxide anion production, ROS generation, NE release, and NET formation in human neutrophils (172). Ginsenoside Rg5 reduces the inflammatory response by inhibiting the activation of the ERK/NF-κB signaling pathway while lowering the cellular Ca^2+^ concentration, thereby suppressing the activity and expression of PAD4 to inhibit NETosis (173).

The potential of these active ingredients to mitigate inflammation by modulating NETs is evident. Modern pharmacological studies suggest that numerous natural products have significant advantages in the treatment of UC (174–178). For example, baicalin was shown to exert its anti-inflammatory effects in UC rats by modulating the IKK/IKB/NF-kB signaling pathway and apoptosis-related proteins (179). Puerarin is one of the major isoflavonoid components of the root of *Pueraria lobata*. In DSS-induced colitis mice, it was demonstrated to exert an anti-inflammatory effect via inhibition of MPO activity and down-regulation of NF-κB and pro-inflammatory mediator secretion. It also possesses antioxidant potential as well as improves intestinal epithelial barrier function (180). Berberine, an isoquinoline alkaloid extracted from Coptidis Rhizoma, exerts a therapeutic role in UC that may involve several aspects such as anti-inflammatory, anti-oxidative stress, maintenance of the structure and function of the intestinal mucosal barrier, modulation of intestinal mucosal immune homeostasis, and regulation of intestinal flora (181–184). Interestingly, berberine has been found to suppress the nuclear translocation of NE and subsequent formation of NETs by inhibiting the interaction of LINC00668 with NE, thus exerting its antithrombotic effect in IBD (104).

### 7.5 Compound prescriptions in TCM

Huang Qin Decoction, a traditional Chinese prescription for UC, inhibits colonic neutrophil infiltration, restores the levels of the intestinal tight junction proteins Occludin and ZO-1, and alleviates intestinal inflammation induced by TNF-α, and IL-1β (152). At the same time, it down-regulates the expression of PAD4 and citH3 to inhibit the production of NETs, which in turn inhibits colitis-associated carcinogenesis (152). In addition, Sijunzi Decoction is also likely to treat UC by decreasing the level of intestinal NETs (185). It has also been found to inhibit the expression of pro-inflammatory cytokines (TNF-α, IL-1β, IL-6) while promoting the expression of anti-inflammatory cytokines (IL-10, IL-37, TGF-β). In light of this evidence, we propose compound prescriptions and active ingredients as a promising therapeutic approach for targeting NETs.

In conclusion, various biological agents and drugs seem to inhibit the formation of NET, but the mechanism needs further investigation. The changes in other physical activities during NET inhibition and the consequences of NET inhibition should not be ignored. Indeed, further studies on the balance between NET induction, inhibition, and degradation are necessary to pharmacologically target NET and its compounds without damaging the immune defenses of patients.

## 8 Conclusions and future perspectives

Excessive NETs cause abnormal activation of the body’s immune system, leading to tissue damage. However, increasing evidence suggests that NETs exert their anti-inflammatory effects by capturing, killing, and clearing pathogenic microorganisms. NETs play an important role in the pathophysiology of many diseases, including UC. NETs are closely related to the inflammatory response, disruption of intestinal epithelial barrier function, and thrombotic trend in UC.

Although NETs have long been shown to be involved in UC, their potential role in UC pathogenesis remains elusive. Obviously, it is necessary to explore the multifaceted biology of neutrophils, especially the regulatory mechanisms controlling the formation and degradation of NETs in the context of UC, to expand our understanding of the pathways leading to increased NETs. In particular, little is known about NET clearance in UC.

In summary, the balance between NET generation and clearance is essential for health. And NETs seem to play a dual role in UC. Targeting NETs opens the door to new therapeutic options for UC. Meanwhile, the beneficial effects of moderate amounts of NETs in UC and the serious consequences of excessive inhibition of its formation cannot be ignored. Therefore, the treatment methods for UC need to be carefully balanced rather than eliminating neutrophil responses.

## Author contributions

DL: Conceptualization, Formal analysis, Investigation, Methodology, Software, Writing – original draft. CM: Conceptualization, Formal analysis, Investigation, Methodology, Software, Writing – original draft. YX: Project administration, Supervision, Writing – review & editing. YZ: Project administration, Supervision, Writing – review & editing.
